# Discrepancy in perception of infertility and attitude towards treatment options: Indonesian urban and rural area

**DOI:** 10.1186/s12978-019-0792-8

**Published:** 2019-08-19

**Authors:** Achmad Kemal Harzif, Victor Prana Andika Santawi, Stephanie Wijaya

**Affiliations:** Department of Obstetrics and Gynecology, Universitas Indonesia, Cipto Mangunkusumo National Hospital, Jl. Pangeran Diponegoro no. 71. Kenari, Senen, Kota Jakarta Pusat, Daerah Khusus Ibukota Jakarta, Jakarta, 10430 Indonesia

**Keywords:** Infertility, Urban, Rural, Knowledge, Myth, Attitude, Misperception, Assisted reproductive technology

## Abstract

**Background:**

In Indonesia infertility affects 10–15% of reproductive-age couples. In addition to medical problem, infertility in Indonesia poses significant social problem. Childlessness is often stigmatized as a failure which victimizes couples, moreover the females. Despite the high prevalence, there is no fertility awareness education which further passes down the common myth, misperception, and negative attitude towards infertility treatment in Indonesian society.

**Objective:**

This study aims to reveal the knowledge, myth, and attitude towards infertility, likewise acceptance towards infertility treatment options.

**Method:**

Cross-sectional study using standardized questionnaire was done to 272 individuals consisted of two parallel groups: Jakarta and Sumba representing urban and rural population respectively. Participants were all outpatients above 18 years old who visited the healthcare centers from February 2017 to June 2017.

**Results:**

Knowledge on biological and lifestyle risk factors of infertility among Jakarta and Sumba groups were comparable. However, belief in supernatural causes of infertility is remarkable in Sumba population. There is a common misconception on the use of contraception as risk factors of infertility in both groups.

Half respondents from both groups think infertility is a disease. In Jakarta 93.4% respondents consider both female and male should be investigated for infertility; in Sumba only 55.4% agree while 33.1% consider only female should be investigated. Infertility is an acceptable reason for polygamy for 41.3% respondents in Sumba, with 34.7% blaming maternal side for childlessness.

Most respondents from both groups accept the use of Assisted Reproductive Technology and fertility enhancing drugs as treatment options.

**Conclusion:**

Lack of understanding, misleading myths, and negative attitude towards infertility have been illustrated in the sample population.

## Plain English summary

Infertility is defined as inability to conceive after 1 year of regular unprotected sexual intercourse. As a disease, many treatment efforts have been suggested. However, infertility has a strong social impact in the culturally and socioeconomically diverse Indonesia. This study attempts to compare the perception of infertility from participants living in two different societies: urban and rural area.

The local participants who grew up in urban (151 participants) and rural area (121 participants) were interviewed using questionnaires individually. The participants from these two locations has a different demographic profile.

It was found that regardless of their difference level of education, they have poor knowledge regarding infertility and what are the risk factors. Half of participants in Indonesian rural area still believe that black magic and mythical reasons are the root for infertility. It is also interesting to highlight that regardless of difference in religion and cultural background, participants from both areas tend to victimize women for infertility. It was found that in both societies, it is more acceptable for the husbands to divorce or remarry due to infertile wife than for the wives to divorce or remarry due to infertile husband.

In conclusion, similar views towards infertility and its treatment option can be observed. Devastating social outcome such as divorce is often deemed acceptable in infertile couple for urban and rural society in Indonesia.

## Background

Infertility is classified as a disease by the World Health Organization, thus considered as a condition that should be treated [1]. Medical professionals deem intervention is necessary towards infertile couple due to high burden it causes towards infertile couples, especially for women. Primary infertility is defined as inability to conceive after 1 year of unprotected sexual intercourse with no previous conception. Secondary infertility is the condition where a couple has conceived previously but became unable then.

Infertility is prevalent, affecting 80 million couples of reproductive age worldwide. In Indonesia, the number translates to 21.3% of couples, affecting roughly one in every couple [2]. Stratified medical approach has been established in Indonesia, however its application is poorly evaluated. Fortunately, 90% of infertility cases have an identifiable cause and half will result in pregnancy given the proper treatment [3].

Infertility poses not only as medical problem, but also a social stigma. Several studies have demonstrated the notorious effect of infertility from social point of views [4–6], There is no formal education in Indonesia regarding infertility. This perpetuates passing down of perception towards infertility, including myths, misinformation, and negative attitudes.

Known modifiable risk factors have been well reviewed, with equal contributing factors from each gender [7]. Whether this knowledge has been well distributed in the population remains a mystery. Indonesia is a multi-cultural country with societies having different religious beliefs, education, social and economic background. Infrastructures also differ by a big margin depending on where the societies are geographically located. Availability of facilities and technological difference ultimately would lead to different accessibility of information.

This study hopes to compare and contrast between participants from urban and rural area of Indonesia. The study sought the level of knowledge regarding infertility, attitude towards infertility, social impact of infertility, and attitude towards infertility treatment options.

## Methods

### Site and study design

Two cross sectional surveys were conducted among conveniently sampled individuals above 18 years old. In Jakarta, the first survey was conducted in primary care center in Pasar Rebo, Pekayon, Indonesia (Puskesmas Kecamatan Pasar Rebo) and in tertiary care hospital in Senen, Jakarta, Indonesia (Rumah Sakit Cipto Mangunkusumo). In East Sumba, the second survey was conducted in primary care center in Waingapu, East Sumba, Indonesia (Puskesmas Kota Waingapu). These centers were selected because they were visited by individuals from various social-economic, education and ethnic backgrounds, and thus provide closer representation of the general population.

### Recruitment and interview

All the outpatients who visited the respective healthcare centers from February 2017 to June 2017 and willing to participate were interviewed in private by their own respecting General Practitioners. Informed consent was asked before each interview. Questionnaire forms were filled in by interviewer based on the respondents’ answer. In Sumba, due to some difficulties in understanding Bahasa Indonesia, some outpatients were omitted from the interview.

### Questionnaire

The questionnaire used was based on previously published literature on infertility [4, 8, 9]. Any language and contextual modification to fit local beliefs were previously consulted to obstetrics and gynecology consultant.

The questionnaires were translated to be written in Bahasa Indonesia, the national language of Indonesia. The terms used were meticulously chosen according to the national dictionary to facilitate comprehension of the questions. Each interviewer has been trained by the authors to equalize the meaning of the questions and its implication.

### Data management

The data was entered to Microsoft Excel 2016 and then imported by SPSS IBM v23 for statistical management.

### Ethical consideration

This study been reviewed and given ethical approval from Ethics Committee of the Faculty of Medicine, Universitas Indonesia (ethical approval protocol number: 0634/UN2.F1/ETIK). All subjects had the right to withdraw from the study anytime they wished without giving any explanation. The questionnaire was anonymous and ensured confidentiality of the study participants. Informed consent was obtained from each participant prior to the interview.

## Results

### Demographic profile

Based on a total sample of 272 individuals interviewed (151 in Jakarta and 121 in East Sumba), the difference in demography could be illustrated. Roughly equal male and female participants were included in both places (mean of 43.1% males and 56.9% females). Mean age was younger in Jakarta participants compared to East Sumba (25.5 ± 6.1 years-old vs. 37.6 ± 12.3 years-old) and most participants in Jakarta were unmarried (76.8% vs. 13.2%). Despite the younger age, formal educational level was much higher in Jakarta (98.7% vs. 55.4% has finished high school). The major religion in each area were also different: 75.5% of Muslims in Jakarta and 69.4% of Christians or Catholics in East Sumba. Table 1 shows the summary of demographic profile.

**Table 1:** Demography of interviewed individuals

Variable	Jakarta		East Sumba	
	N	%	N	%
Sex				
Male	64	42.4%	53	43.8%
Age				
18–20	3	2.0%	2	1.7%
20–29	129	85.4%	33	27.3%
30–39	13	8.6%	43	35.5%
40–49	4	2.6%	22	18.2%
50–59	1	0.7%	11	9.1%
> 59	1	0.7%	10	8.3%
Marital Status				
Unmarried	116	76.8%	16	13.2%
Married	34	22.5%	100	82.6%
Divorced	1	0.7%	1	0.8%
Widow/er	0	0.0%	4	3.3%
Education				
None	0	0.0%	9	7.4%
Primary	0	0.0%	26	21.5%
Secondary	2	1.3%	19	15.7%
High school	24	15.9%	41	33.9%
Diploma	7	4.6%	8	6.6%
Bachelor	118	78.1%	18	14.9%
Religion				
Muslim	114	75.5%	36	29.8%
Christian	17	11.3%	58	47.9%
Catholic	12	7.9%	26	21.5%
Buddha	3	2.0%	0	0.0%
Hindu	0	0.0%	0	0.0%
Others	5	3.3%	1	0.8%

### Knowledge of infertility

The question regarding knowledge of the participants preceded by the definition of infertility. None of the participants in both areas could correctly define infertility as inability of conceive after 1 year of unprotected sex. In addition, the term sterile was often confused with infertile.

The next set of questions addressed the risk factors for infertility. Several identified factors and common myths were then read towards the participants and they would decide whether certain factors were causing infertility. The similarities and contrasts between both groups can be seen in Table 2.

**Table 2:** Knowledge on risk factors of infertility

Knowledge on risk factors of infertility	Jakarta		East Sumba	
	N	%	N	%
Menstrual disturbance	82	54.3%	59	48.8%
Problem in uterine passage	116	76.8%	68	56.2%
History of genital tract infection in females	63	41.7%	48	39.7%
History of genital tract infection in males	57	37.7%	43	35.5%
Smoking	97	64.2%	73	60.3%
Obesity	72	47.7%	61	50.4%
Psychological stress	113	74.8%	78	64.5%
History of Oral Contraceptive Pills	59	39.1%	63	52.1%
History of Intrauterine device	41	27.2%	57	47.1%
Mythical or supernatural causes	7	4.6%	63	52.1%
Black magic	9	6.0%	64	52.9%
Regular exercise	6	4.0%	22	18.2%

The knowledge for both groups regarding the medical risk factors for infertility were poor. Less than half participants could identify all medically related risk factors of infertility. Both groups were also unaware of social and lifestyle factors of infertility. Roughly half participants were misled in the danger of smoking, obesity, and psychological stress (as can be seen in Table. 2).

There was an interesting finding in the knowledge regarding the use of medically approved birth control on long term infertility. In Jakarta, more people believed that birth control would not affect long-term fertility. More than one third (39.1%) of participants in Jakarta still identify the use of Oral Contraceptive Pill (OCP) to cause long-term infertility, and more so (52.1%) in East Sumba. Similar trend could be observed in the perceived effect of Intrauterine Device (IUD) use. More than one fourth (27.2%) of participants in Jakarta and roughly half participants (47.1%) in East Sumba thought that IUD causes infertility.

One major difference was found with regards to supernatural belief in fertility. In East Sumba, more than 50% of the participants had superstition that mythical or black magic contribute to infertility, while only roughly 5% of participants agreed in Jakarta.

Overall, when comparing the data qualitatively, there was a higher percentage of East Sumba participants who were misinformed. In East Sumba, more participants seemed to view contraception (OCP and IUD) as a mean to permanently reduce infertility when compared to actual medically related risk factors (Menstrual disturbance, Problem in uterine passage, and history of genital tract infection). This trend was not observed among participants in Jakarta who seemed to be lacking information rather than misinformed regarding causes and risk factors of infertility.

### Attitude towards infertility

Half of participants in both groups identified infertility not as a disease (56.3 and 49.6% among participants in Jakarta and East Sumba respectively). However, upon given the next question regarding the necessity for treatments for infertile couple, majority of participants would agree. Most participants in Jakarta agreed to treat infertile couple, while one in five participants considered that infertility does not need to be treated in East Sumba (96.0% vs. 81.8%). Among those believing that infertility does not need to be treated, many states that infertility is something congenital and already destined from the beginning by God or genetic.

The next question assumed that an infertile couple would like to be investigated and participants were asked about who should be examined first. Majority of the participants in Jakarta answered that both husband and wife should be investigated. One third of participants in East Sumba thought that women should be investigated first.

Later question addressed the possibility of secondary infertility. Participants in Jakarta were less well informed with only one third acknowledges secondary infertility, while more than half in East Sumba claimed that it was possible to become infertile even after conceiving (36.4% vs 57.0%).

The last question assessed where the participants would seek treatment for infertile couple. They were given options to consult medical doctors, witch doctors, self-medicate, or seek other alternative therapies. The differences between two groups were distinct. While only one participant in Jakarta would like to seek other help before consulting medical doctors, 36.4% of participants in East Sumba preferred to seek help from other alternatives first. The most popular alternative therapy was traditional massage in the lower abdominal and lower back area.

Given failure of the preferred initial treatment, 49.0% of participants in Jakarta and 34,7% of participants in East Sumba were still confident to consult another medical doctor. However, there were some participants in East Sumba that would not seek help from medical doctors (11.6%) even when alternative therapies had failed. The participants claimed that this was due to unavailability of medical doctors and perceived incompetence of medical doctors in treating infertility (Table 3).

**Table 3:** Attitude towards infertility

Attitude towards infertility	Jakarta		East Sumba	
	N	%	N	%
Infertility regarded as a disease				
Yes	85	56.3%	60	49.6%
Treatment is needed for infertile couple				
Yes	145	96.0%	99	81.8%
Who should be first investigated among infertile couples				
Husband	3	2.0%	14	11.6%
Wife	7	4.6%	40	33.1%
Both	141	93.4%	67	55.4%
Possibility of secondary infertility				
Yes	55	36.4%	69	57.0%
First preference for treatment				
Doctors	150	99.3%	77	63.6%
Others	1	0.7%	44	36.4%
Secondary preference for treatment				
Doctors	74	49.0%	42	34.7%
Others	77	51.0%	79	65.3%

### Treatment options

There were more participants approving divorce in the event of infertility in East Sumba (27.3% for infertile female and 16.5% for infertile male). In contrast, participants in Jakarta seemed to be more resilient in their marriage when encountering infertility, with less participants who approved divorce (4.0% for infertile female and 7.3% for infertile male).

Similar trend was observed for approving the husbands to remarry in the event of infertile female: 41.3 and 17.2% of participants in East Sumba and Jakarta respectively. However, in the case of infertile male, both groups seemed to be less approving for the wives to remarry: 18.2 and 11.9% of participants in East Sumba and Jakarta respectively.

In addition, both groups claimed that the wives were being blamed by the society in the event of infertility. Majority of the participants in Jakarta (59.6%) claimed that neither can be blamed, while only roughly one third of participants (38.0%) in East Sumba agreed. Among the participants claiming that the society was blaming the infertile couples, 34.7 and 23.8% in East Sumba and Jakarta claim that the wives were the victim. In contrast, less participants claimed that the husbands were the victim: 7.4 and 1.3% in East Sumba and Jakarta respectively.

Adoption was favorable in both groups and viewed as a good solution for infertile couples. Most participants would approve adoption as long as full consent from both biological parents and the infertile couple could be achieved (83.4 and 84.3% in Jakarta and East Sumba respectively).

Preceding the next question, a little explanation on “test-tube baby” was given due to lack of knowledge on the subject. It was defined as taking the sperm and egg of the infertile couple, followed conducting fertilization outside the body, and transferring the embryo back to the biological mother so that she could bear child normally. The use of surrogate mother for the procedure was not the intention of this question. Majority of the participants in both groups seemed to be able to accept the idea (82.1 and 74.4% in Jakarta and East Sumba respectively). Those participants disagreeing to such procedure would claim that it was not natural and as though humans were playing God. However, most participants would accept the use of fertility enhancing drug (92.1 and 96.7% in Jakarta and East Sumba respectively). The small percentage who disagreed would claim that it was unnecessary to treat infertility altogether because if it was the will of God that a couple is infertile (Table 4).

**Table 4:** Options for infertile couples

Options for infertile couples	Jakarta		East Sumba	
	N	%	N	%
Infertile female: divorce				
Yes	6	4.0%	33	27.3%
Infertile female: remarry				
Yes	26	17.2%	50	41.3%
Infertile male: divorce				
Yes	11	7.3%	20	16.5%
Infertile male: remarry				
Yes	18	11.9%	22	18.2%
Blamed by the society				
Husband	2	1.3%	9	7.4%
Wife	36	23.8%	42	34.7%
Both	23	15.2%	24	19.8%
Neither	90	59.6%	46	38.0%
Adoption				
Yes	126	83.4%	102	84.3%
Use of Test-tube Baby				
Agreeable	124	82.1%	90	74.4%
Use of Fertility drug				
Yes	139	92.1%	117	96.7%

## Discussion

Despite diverse demography of participants in Jakarta and Sumba, there is low level of knowledge towards fertility. Regardless of difference in age, level of education, marital status, and religion, participants from both groups have low knowledge about infertility. The higher level of education nor better access of information in Jakarta does not prevent naivety towards risk factors of infertility. This shows that participants are unaware how to prevent infertility and when or where to seek help should they are infertile. This tendency is also observed globally in a survey in the World Fertility Awareness Month [10]. This may be explained by the lack of basic reproductive health education in Indonesia.

Furthermore, the participants are not merely uninformed about infertility, but rather they have erroneous knowledge. One interesting finding is that roughly half of participants do not believe the advertisement and medical professionals’ advice on the danger of smoking that may harm fertility [2]. Many participants argue that they know many who smokes yet having no problem to conceive. The same trend can be observed in obesity and psychological stress as modifiable risk factors. These lifestyle risk factors are regarded of less importance in influencing one’s fertility. This suggest that in cases of infertility, participants would often overlook these modifiable risk factors.

Paradoxically, advices by medical professionals on the use of contraceptive pills and intrauterine device to withheld pregnancy is often believed to permanently decrease fertility. This finding suggests lack of knowledge on how contraception works as participants may falsely blame contraception for infertility. This lack of understanding will lead to several socio-economic problems associated with averting birth control: overpopulation and poverty [11]. A survey has shown that 15.5% of married reproductive age women in Indonesia has never undergone any contraception, despite enthusiastic campaign by the Government [12]. This is contrary to a survey by Mac Dougall et al. on 61 women who visited fertility clinics in the USA, in which fertility was overestimated, hence 23% stressed lifelong pregnancy control using contraceptive methods. While our study subjects in general fear of fertility decline following contraceptive use, subjects in Mac Dougall et al. study perceived the need to “control” pregnancy in fear of unexpected pregnancy following cessation of contraception [13].

Another erroneous perception would be assuming that infertility is not a disease. The participants tend to view infertility as something absolute or congenital. If anything, infertility is often associated as the will of higher being. Thus, it becomes a habit among them to seek more spiritual approach to solve infertility. This view is complementary to their obliviousness towards secondary infertility. Participants tend to think that once a couple can conceive, it is simply about time and other superstitious factors that will conceive again. The unfavorable outcome from this misperception would lead to couples not seeking treatment.

Level of trust towards medical professionals in East Sumba, which is rural area, is surprisingly low. Rather, some of the participants show blind faiths towards alternative healing. Although almost all participants have a religion, many also believes in the power black magic and shaman. This is consistent with previous findings on low level of knowledge on infertility. It then becomes a common practice to visit the shamans before medical professionals when faced with infertility. A prospective study on urban population in the US by Smith et al. identified 45% of couples seeking fertility care had tried alternative fertility treatments such as acupuncture, herbal, body work and meditation [14]. This means that alternative treatment across various socio-economic and educational background is always a considerable option.

The problem is even greater in our society in which our finding revealed some of the participants would rather not visit medical professionals at all. This might be explained by higher availability of shamans compared to doctors, also perpetuated by gap in availability of medical amenities and trained medical doctors in Jakarta and East Sumba. This is consistent with previous statement regarding unequal availability of medical competence and technologies across Indonesia [2]. Eventually, several members of the community, especially those living in rural areas have inadequate fertility treatment.

The outcome of infertility is indeed devastating. Victimization towards infertile couples occurs regardless of religious beliefs and socio-economical background. Although not allowed by religions in Indonesia, the pressure from the society towards infertile couple may consequently lead to divorce. Despite equal contribution of male and female factors to infertility cases, women seem to suffer more from the disease both in terms of social stigma and divorce. The similar trend can be observed in three different countries: Saudi [15], Iran and Pakistan [4]. This may be due to lack of knowledge regarding the medical causes and infertility treatment options observed among developing countries.

Adoption might offer a better solution than divorce. Most participants in both groups would agree to adopt a child for infertile couples. From the participants response we can infer that people think married couple should have child, regardless of bearing their own or adopting one. However, adoption has not become a common practice. The data in Indonesia is lacking, but in Karachi, capital city of Pakistan, only 6% of 400 infertile couples have adopted a child [5]. There seems to be more benefit for infertile couples to adopt a child, but it has high complexity and remains debatable from psychological perspectives [16]. The seemingly low trend of infertile couple opting for adoption may suggest that particularly for childless couple, adopting a child is inferior and cannot replace the feeling of having one on their own [17].

Assisted Reproductive Technology (ART) might offer a better solution for infertile couples [18]. The term of “test-tube baby” is often unfamiliar among the participants. During our survey we observed the common trend of fear of the unknown and assumption of playing God caused, the participants to be reluctant towards the idea. This was alleviated when short explanation of the procedure is explained to the participants. Thus, in the result we can see most participants agree towards the use ART to combat infertility. Similar findings has been observed in the presence of gap due to lack of knowledge between the potential and willingness of ART procedures [19]. This suggests that better understanding towards infertility and how ART works will improve its acceptance in both urban and rural regions.

The mean of understanding fertility seems to occur through mouth-to-mouth conversations between laymen in Indonesia. This explains why erroneous information tends to spread in the community. Similar problem was also reported by Mac Dougall in which incorrect information spread among the subjects mostly through friends, misleading media reports, and even physicians in the United States [13].

Despite our attempt to provide data representing urban and rural population in Indonesia, our study has its limitations in terms of its representativeness. Total sampling method used to recruit respondents may lead to poor representation of the population. Although participants in Jakarta were recruited in both primary healthcare center and tertiary care hospital, heterogeneity is still lacking. Subjects in Jakarta greatly represent under 30-year-old age group (87.4%), unmarried (76.8%), and higher educated subjects. This may cause the researchers to overlook the subset of subjects who were married, older and with lower educational background. We also did not incorporate income groups in our questionnaire. Previous studies by Nachtigall et al. have demonstrated that decision to seek fertility treatment, more specifically ART that is notoriously expensive is highly influenced by economical background [20].

## Conclusions

Despite difference in demography between the two groups, similar views towards infertility and its treatment option can be observed. Ultimately, both groups have low level of knowledge, negative attitude, and false perception towards infertility. Devastating social outcome such as divorce is often deemed acceptable in infertile couple. Short information on infertility and its treatment has been shown to increase acceptability towards medical interventions. This study can hopefully be used as ground for future fertility education with regards to cultural competence.

## Data Availability

The datasets generated and/or analyzed during the current study are available in the google sheets repository: https://docs.google.com/spreadsheets/d/13OqZYqPED5XZhrHVqct9Hf8WMoK8T3kj6DJJYSLrOrE/edit. The datasets generated and/or analyzed during the current study are not publicly available due to ethical concern of personal information regarding socio-economic status and religious beliefs but are available from the corresponding author on reasonable request.

## Abbreviations

ART: Assisted Reproductive Technology
IUD: Intrauterine Device
OCP: Oral Contraceptive Pills
